# Kinetics of prostate volume reduction under hormonal treatment: Timing and impact on external beam radiotherapy – A PRISMA-Compliant systematic review

**DOI:** 10.1016/j.ctro.2026.101129

**Published:** 2026-02-17

**Authors:** Youssef Ghannam, David Pasquier, Mario Terlizzi, Paul Sargos, Bertrand Tombal, Vérane Achard

**Affiliations:** a Department of Radiation Oncology, Gustave-Roussy, Villejuif, France; b Academic Department of Radiation Oncology, Oscar Lambret Center, Lille, France; c University of Lille, CRIStAL UMR 9189, Lille, France; d Department of Radiotherapy, Bergonie Institute, Bordeaux, France; e Amethyst Radiotherapy Group, Paris, France; f Department of Urology, Cliniques Universitaires Saint-Luc, Université catholique de Louvain (UCLouvain), Brussels, Belgium; g University of Geneva, Geneva, Switzerland

## Abstract

**Purpose:**

Understanding how hormonal treatment affects prostate volume is essential for optimizing external beam radiotherapy (EBRT) planning in localized prostate cancer. The study aimed to characterize the kinetics of prostate volume reduction during hormonal therapy and assess its implications for EBRT delivery and planning strategies.

**Methods and materials:**

A systematic review following PRISMA guidelines was conducted. Clinical studies assessing prostate volume changes under hormonal therapy in non-metastatic prostate cancer patients were included. Imaging modalities (MRI, CT, TRUS) were used to track prostate volume over treatment durations of up to 25 months. The review excluded preclinical studies and salvage therapy cases. Data were synthesized descriptively due to heterogeneity in regimens and methodologies.

**Results:**

Across 35 studies, prostate volume followed a logarithmic decay under hormonal treatment, with the most significant reductions occurring in the first three months (up to 35%). The extent of volume reduction during EBRT varied by EBRT initiation timing and fractionation scheme, with greater changes observed in normofractionated regimens. A weak correlation was found between initial prostate volume and reduction rate. Limitations included heterogeneity in imaging protocols and lack of formal bias assessment.

**Conclusions:**

Prostate volume reduction is most pronounced early in hormonal treatment, emphasizing the need to synchronize imaging and EBRT planning. For high-precision techniques (e.g., SBRT or DIL boosting), accounting for volume kinetics is critical. Clinical practice should evolve toward integrating prostate volume reduction timelines into EBRT workflows.

## Highlights

• Prostate volume reduction follows a predictable logarithmic decay under hormonal treatment.
• Up to 35% shrinkage occurs within the first three months of hormonal therapy.
• Radiotherapy planning must account for hormonal treatment-induced prostate downsizing.

## 1 Introduction

Extensive research provides robust evidence that androgen deprivation therapy (ADT) increases the efficacy of external beam radiotherapy (EBRT) in patients with intermediate and high-risk localised prostate cancer. The Meta-Analysis of Randomized trials in Cancer of the Prostate (MARCAP) Consortium, an individual patient data (IPD) *meta*-analysis of 12 randomised trials, involved 10,853 patients with a median follow-up of over 11 years [1]. MARCAP confirmed that ADT, when combined with EBRT, significantly improved biochemical recurrence (BCR), metastatic progression, metastasis-free survival (MFS), and overall survival (OS), compared to EBRT alone. The benefits were observed regardless of EBRT dose, patient age, or risk group. For patients with intermediate-risk disease, a short duration of 4–6 months of ADT appears optimal [2], while those with high-risk disease benefit from extended durations of 2–3 years [3].

ADT induces the apoptosis of benign and malignant prostate cancer cells, thus leading to a significant volume reduction of the gland [4]. ADT-induced prostate volume reduction has been known for more than 30 years. Brachytherapy is frequently associated with a short course of neoadjuvant ADT to facilitate seed implantation[5]. The extent to which volume reduction is considered in EBRT planning is less well defined. The ESTRO ACROP guidelines on CT- and MRI-based target volume delineation for EBRT of localised prostate cancer do not specify a special timing across the ADT course to plan the patient’s treatment course [6]. As for the guidelines on image-guided radiation therapy (IGRT), they indeed mention that neoadjuvant ADT induces prostate deformation, but make no specific further recommendations about how to take into account this volume variations during planning and treatment course [7].

Taking into consideration prostate volume reduction can be critical when using modern EBRT techniques that depend on precise delineation of the prostate and nearby organs at risk (OARs). For radiation oncologists who still use normofractionated schedules, the EBRT course can span nearly 2 months [8]. During this period, the prostate shrinks, resulting in a different anatomical setup at the end of treatment compared to the start. Such changes can increase OARs exposure to high radiation doses [9], potentially leading to increased toxicity [10]. Furthermore, as EBRT techniques become more accurate, especially in the detailed delineation of urinary OARs (e.g., bladder substructures and urethra) [11] and employing focal boosting targeting dominant intraprostatic lesions (DIL) mostly MRI-defined [12], [13], it becomes essential to consider prostate volume changes. Prostate volume reduction under ADT may notably alter the spatial relationships between targets and nearby OARs, potentially invalidating pre-ADT dosimetry plans. Therefore, understanding prostate volume changes during ADT is crucial for timing MRI acquisition to achieve precise contouring.

This systematic review aims to characterise the time-dependent pattern of prostate volume reduction and examine its practical implications for EBRT planning and delivery, especially in the context of moderately hypofractionated EBRT, ultra-hypofractionated EBRT (SBRT) and DIL boosting.

## 2 Evidence acquisition

### 2.1 Study design

This systematic review was conducted according to the Preferred Reporting Items for Systematic Reviews and Meta-Analyses (PRISMA) statement. The primary objective was to synthesise the evidence on the kinetics of prostate volume reduction under hormonal treatment and to discuss the potential implications for EBRT planning.

### 2.2 Search strategy

A comprehensive search of the PubMed database was performed without date restrictions. The following predefined search string was used:

((androgen deprivation[Title/abstract]) OR (antiandrogens[Title/abstract]) OR (degarelix[Title/abstract]) OR (goserelin[Title/abstract])) AND (prostate[Title/abstract]).

AND ((volume[Title/abstract]) OR (lower urinary tract symptoms[Title/abstract]) OR (LUTS[Title/abstract])) NOT ((metastatic[Title/abstract]) OR (metastases[Title/abstract]) OR (oligometastatic[Title/abstract]) OR (castration resistant[Title/abstract]) OR (salvage [Title/abstract]) OR (recurrent[Title/abstract]) OR (mouse[Title/abstract]) OR (mice[Title/abstract])).

The search was restricted to English-language articles. The last search was performed on 15 May 2025.

### 2.3 Eligibility criteria

We included prospective and retrospective clinical studies assessing prostate volume changes under hormonal treatment. Eligible studies needed to use imaging to measure prostate volume at baseline and throughout hormonal treatment follow-up. We excluded studies involving metastatic, oligometastatic, castration-resistant, recurrent prostate cancer or salvage treatment populations and preclinical studies. No minimum sample size was specified.

### 2.4 Study selection

Two independent reviewers (YG and VA) screened titles and abstracts for eligibility. The full texts of potentially relevant studies were then assessed for the inclusion criteria. Any discrepancies between reviewers were resolved by consensus after discussion. Duplicate records were removed before screening.

### 2.5 Data extraction and quality assessment

For each included study, we extracted data on study design, number of patients, baseline patient and disease characteristics, hormonal treatment regimen and duration, imaging modality used for volume assessment, baseline and follow-up prostate volumes, absolute and relative changes in volume, and timing of measurements. Data were compiled in a structured extraction table to describe the kinetics of prostate volume change under hormonal treatment. No formal risk-of-bias assessment was performed, as the primary aim was descriptive synthesis.

### 2.6 Data synthesis and statistical analysis

Due to heterogeneity in study design, patient populations, hormonal treatment regimens, imaging protocols, and follow-up intervals, a quantitative *meta*-analysis was not feasible. Data are presented descriptively, with a focus on mapping the reported temporal patterns of prostate volume reduction and identifying relevant time points for potential EBRT replanning. For modelling the temporal kinetics of prostate volume reduction, summary prostate volume values (mean or median, as reported in the original studies) were extracted at available time points and pooled into a descriptive dataset. Because individual patient data were unavailable and reporting formats differed across studies, values were not standardized and data points were not weighted by study sample size. The resulting logarithmic model should therefore be interpreted as an illustrative descriptive fit rather than a formal *meta*-analytic estimate.

## 3 Evidence synthesis

### 3.1 Study selection and characteristics

**Fig. 1:**
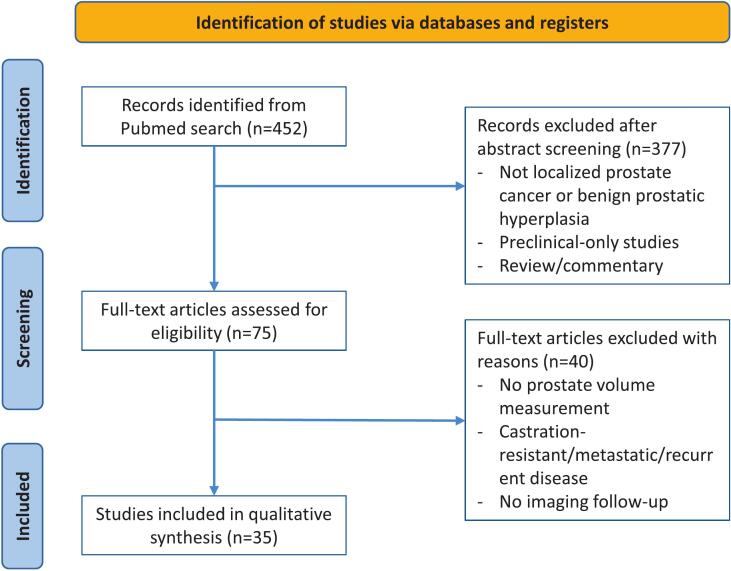
Flowchart of included studies. *n; numbers.*

A total of 35 clinical studies met the inclusion criteria and were included in this systematic review (Fig. 1) [9], [14], [15], [16], [17], [18], [19], [20], [21], [22], [23], [24], [25], [26], [27], [28], [29], [30], [31], [32], [33], [34], [35], [36], [37], [38], [39], [40], [41], [42], [43], [44], [45], [46], [47]. Various imaging modalities (TRUS, CT, MRI) and hormonal treatment regimens (LHRH agonists, antagonists, or combined) were reported, with follow-up periods ranging from 1 to 12 months. Most studies recorded volume measurements at a single time point before and during treatment (Table 1). All studies but one [16] focused on patients treated for localised prostate cancer.

**Table 1:** Volumetric and Clinical Data.

**Year, Name**	**N**	**Volume evaluation technique**	**Type of hormonal treatment**	**Vol1 (mL), mean or median**	**T2 (mo)**	**Vol2(mL)**	**Vol2 (%)**	**T3 (mo)**	**Vol3(mL)**	**Vol3(%)**
**1993, Schulman**[14]	40	TRUS	LHRH agonist + flutamide	X	3.0	NS	50–60	NS	NS	NS
**1999, Whittington**[15]	69	TRUS	Leuprolide or Goserelin	40.0	3.4	29.6	67.0	NS	NS	NS
**1993, Tempany**[16]	20	MRI	Finasteride	61.7	12.0	50.1	69.0	NS	NS	NS
**1994, Voges**[17]	70	TRUS	Goserelin + flutamide	X	3.0	NS	69	NS	NS	NS
**1996, Chen**[18]	10	MRI	NS	X	3.2	NS	66.5	NS	NS	NS
**1996, Polito**[19]	62	TRUS	LHRH agonist + flutamide	34.9	3.0	26.9	76.8	NS	NS	NS
**1996, Tunn**[20]	375	TRUS	cyproterone acetate, LHRH agonist + antiandrogen (cyproterone acetate or flutamide)	29.8	2.0	21.4	71.8	NS	NS	NS
**2000, Lilleby**[21]	13	CT/MRI	Goserelin + Cyproterone acetate	56	1.0	46.0	82	3.0	37.5	67.0
**2002, Kucway**[22]	63	TRUS	Leuprolide + bicalutamide/nilutamide/flutamide	53.6	3.7	34.8	65.0	NS	NS	NS
**2002, Kucway**[22]	44	TRUS	Leuprolide or Goserelin	53.6	3.7	37.5	70.0	NS	NS	NS
**2003, Henderson**[23]	19	TRUS	Goserelin + 2 wk antiandrogen (flutamide or cyproterone acetate)	38.0	3	30.0	79.0	NS	NS	NS
**2003, Henderson**[23]	31	TRUS	Bicalutamide	33.7	3	31.3	93	NS	NS	NS
**2004, Merrick**[24]	101	TRUS	LHRH agonist + antiandrogen	61.8	3.8	39.3	63.6	NS	NS	NS
**2006, Niehaus**[25]	418	TRUS	LHRH agonist + antiandrogen	34.2	4	25	73	NS	NS	NS
**2007, Ebara**[26]	91	TRUS	Leuprolide or Goserelin	35.6	4.8	24.2	68.0	NS	NS	NS
**2007, Ebara**[26]	49	TRUS	Bicalutamide or Flutamide	26.4	6.2	21.6	81.9	NS	NS	NS
**2007, Ebara**[26]	48	TRUS	LHRH agonist + antiandrogen	49.7	6.1	29.2	58.8	NS	NS	NS
**2007, Petit**[28]	42	TRUS	Leuprolide	54.0	3	34.0	70.0	NS	NS	NS
**2007, Petit**[28]	14	TRUS	Goserelin	54.0	3	34.0	70.0	NS	NS	NS
**2007, Petit**[28]	25	TRUS	Bicalutamide	54.0	3	34.0	70.0	NS	NS	NS
**2008, Petit**[27]	58	TRUS	Leuprolide or Goserelin	55	3	34.0	68.0	NS	NS	NS
**2008, Petit**[27]	25	TRUS	Bicalutamide	50	3	34.0	74.0	NS	NS	NS
**2010, Padhani**[29]	23	MRI	LHRH agonist and/or antiandrogens	42	3.6	24.7	50	NS	NS	NS
**2011, Langenhuijsen**[30]	20	CT	Buserelin + Nilutamide	82	3.0	58.0	70.7	6.0	52.0	63.4
**2012, Axcrona**[31]	92	TRUS	Goserelin	49.9	3.0	NS	61	NS	NS	NS
**2012, Axcrona**[31]	81	TRUS	Degarelix	54.8	3.0	NS	62.8	NS	NS	NS
**2012, Klarskov**[32]	77	TRUS	Orchiectomy (n = 10), LHRH agonist (n = 6), Antiandrogen (n = 55), Combination (n = 6)	43	3.0	27.0	62.8	12.0	25.5	59.3
**2013, Melancon**[33]	44	CT	Leuprolide + Bicalutamide	56.7	2.0	42.7	75.7	NS	NS	NS
**2013, Mason**[34]	57	TRUS	Goserelin	52.5	3.0	NS	64.7	NS	NS	NS
**2013, Mason**[34]	164	TRUS	Degarelix	50.9	3.0	NS	64	NS	NS	NS
**2013, Anderson**[35]	13	TRUS	Goserelin + 2 wk bicalutamide	50.3	3.0	NS	75	NS	NS	NS
**2013, Anderson**[35]	27	TRUS	Degarelix	53.5	3.0	NS	58	NS	NS	NS
**2014, Kim**[36]	48	MRI	Bicalutamide (4), Bicalutamide + Goserelin (5), Bicalutamide + Triptorelin (12), Bicalutamide + Leuprolide (9), Bicalutamide + Goserelin + Triptorelin(10), Bicalutamide + Goserelin + Triptorelin + Leuprolide (2)	42.8	2.0	21.4	50	NS	NS	NS
**2015, Hötker**[37]	30	MRI	LHRH agonist and/or antiandrogen	57	4.0	39.0	67	NS	NS	NS
**2016, Choi**[38]	110	TRUS	Leuprolide + Bicalutamide	36.7	20.4	19.5	53.2	NS	NS	NS
**2016, Washino**[39]	32	TRUS	Goserelin + Bicalutamide	29.5	3.0	NS	62.5	6.0	NS	52.5
**2018, Korzeniowski**[40]	46	TRUS	Degarelix	65.0	2	48.2	73.8	NS	NS	NS
**2018, Hayama**[41]	29	TRUS + transabdominal US	Leuprolide + Bicalutamide	41.5	6.0	27.0	65.8	NS	NS	NS
**2019, Yikilmaz**[42]	24	TRUS	Goserelin	64.1	6.0	NS	79.8	NS	NS	NS
**2019, Yikilmaz**[42]	27	TRUS	Leuprolide	81.2	6.0	NS	84.4	NS	NS	NS
**2020, Christie**[43]	50	MRI	Triptorelin	45.6	1.5	35.2	77.2	3.0	28.9	63.7
**2020, Dearnaley**[44]	65	TRUS or MRI	Relugolix	36.5	2.5	25.6	73.7	NS	NS	NS
**2020, Dearnaley**[44]	38	TRUS or MRI	Degarelix	40	2.5	29.7	70.9	NS	NS	NS
**2021, Bjøreland**[45]	50	MRI	LHRH agonist	61.5	3.0	36.4	59.2	6.0	31.0	50.4
**2021, Tharmalingam**[46]	37	MRI	Leuprolide	67.1	3.0	44.7	66.6	NS	NS	NS
**2023, Merten**[9]	17	MRI	NS	88.3	4.3	66.4	75.2	NS	NS	NS
**2025, Fukuokaya (SHIP0804) – AHT arm**[47]	198	TRUS	Goserelin or Leuprolide	29.5	3	22.5	76.0	NS	NS	NS
**2025, Fukuokaya (SHIP0804) – non-AHT arm**[47]	205	TRUS	Goserelin or Leuprolide	29.8	3	22.7	76.	NS	NS	NS

MRI = Magnetic Resonance Imaging; TRUS = Transrectal Ultrasound; LHRH = Luteinizing Hormone-Releasing Hormone; CT = Computed Tomography; US = Ultrasound;

PSA = Prostate-Specific Antigen; NS = Not Specified; wk = week(s); mo = month(s); Vol1-3 = Prostate volume at baseline, after T2 and T3; T2-3 = Time points; AHT = Adjuvant Hormonal Therapy.

### 3.2 Prostate volume Reduction: Kinetics across studies

**Fig. 2:**
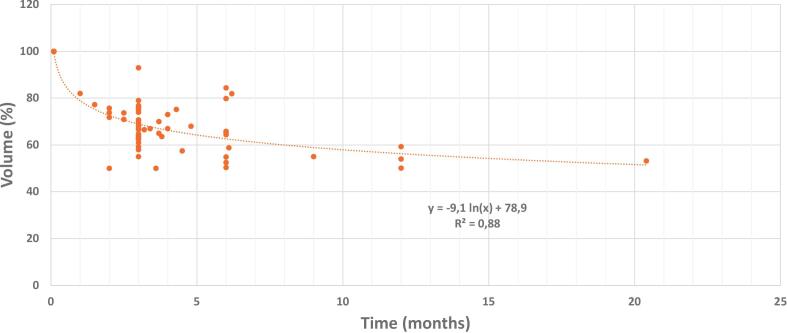
Temporal kinetics of prostate volume reduction under hormonal treatment.

The overall temporal pattern of prostate volume reduction during hormonal treatment was effectively modelled over a 25-month observation period (Fig. 2). The decrease in volume closely followed a logarithmic decay trend, represented by the equation y =  − 9.1·ln(x) + 78.9, where x denotes time in months. To accommodate the mathematical constraint that the natural logarithm is undefined at zero, baseline time points recorded at 0 months were shifted to 0.1 month. This allowed for continuous curve fitting while preserving the interpretability of early treatment kinetics. The model demonstrated a strong fit to the aggregated data (R^2^ = 0.88). Importantly, analysis of this logarithmic function indicates that the highest rate of volume reduction occurs in the first three months of hormonal treatment. This rapid change corresponds to the steepest slope of the curve at the earliest time points (when Time *x* is smallest), suggesting that the greatest change in volume per unit time occurs during the initial phase of hormonal treatment.

### 3.3 Impact of initial prostate volume

**Fig. 3:**
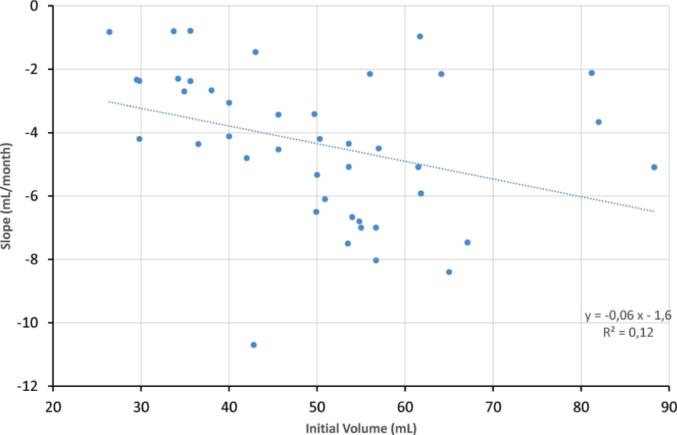
Prostate volume decrease versus initial prostate volume.

The influence of initial prostate volume (mL) on the subsequent instantaneous rate of volume reduction (Slope, mL/month) was investigated (Fig. 3). A linear correlation was observed, defined by the equation y =  − 0.06x − 1.6. However, this model demonstrated a weak predictive relationship, with a low coefficient of determination (R^2^ = 0.12). This suggests that the initial volume, while potentially exhibiting a slight trend toward a greater negative slope (faster reduction) with larger starting volumes, is not a strong independent predictor of the rate of volume change.

### 3.4 Impact of EBRT timing and fractionation

**Fig. 4:**
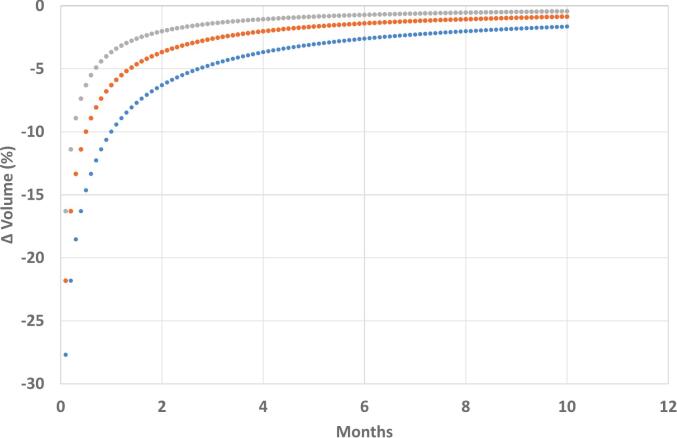
Prostate volume decrease during the entire course of radiotherapy, according to the timing of RT initiation after ADT, for the normofractionated schedule (2 months, blue curve), the moderately hypofractionated schedule (1 month, orange curve), and the SBRT schedule (0.5 months, grey curve). (For interpretation of the references to colour in this figure legend, the reader is referred to the web version of this article.)

The percentage change in prostate volume (Δ Volume (%)) occurring during EBRT was evaluated as a function of the delay between hormonal treatment initiation and EBRT start (Fig. 4). Three distinct fractionation schedules were modelled based on their treatment duration: normofractionated treatment (Curve 1: *y*(2 + *x*) − *y*(*x*)), lasting 2 months; moderately hypofractionated treatment (Curve 2: *y*(1 + *x*) − *y*(*x*)), lasting 1 month; and SBRT (Curve 3: *y*(0.5 + *x*) − *y*(*x*)), lasting 2 weeks (0.5 months).

Across all three treatment regimens, the volume reduction achieved during the EBRT interval was highly dependent on the timing of EBRT initiation. The maximal reduction occurred when EBRT was initiated earliest, with the normofractionated treatment yielding the most significant decrease, approaching 35%. As the initiation of EBRT was progressively delayed (up to 12 months after the start of hormonal treatment), the magnitude of the Δ Volume (%) achieved during the EBRT phase rapidly decreased, approaching zero.

When comparing treatment regimens, longer duration schedules consistently resulted in a greater percentage volume reduction during the defined EBRT period. For example, delaying EBRT initiation until two months after hormonal treatment commencement still showed a clear duration-dependent effect: the 2-month normofractionated schedule resulted in a volume reduction of approximately − 16% to − 17%; the 1-month moderately hypofractionated schedule yielded an intermediate reduction of − 12% to − 13%; and the 0.5-month SBRT schedule resulted in the smallest immediate reduction, approximately − 8% to − 9%. This demonstrates that, while delaying the start of EBRT diminishes the overall volume reduction benefit relative to immediate initiation, the intrinsic duration of the EBRT schedule remains a determinant of volume change during the treatment window.

## 4 Discussion

Prostate volume reduction resulting from ADT has been well-documented for decades. However, its implications have been somewhat underappreciated over the years by clinicians administering EBRT for prostate cancer. This consideration remains highly relevant today, particularly in light of recent findings from the MARCAP *meta*-analysis on ADT sequencing [48] and the increasing adoption of DIL boosting strategies.

The MARCAP *meta*-analysis demonstrated that, for patients receiving six months of ADT alongside prostate-only EBRT, a concurrent/adjuvant ADT schedule was associated with improved MFS and reduced prostate cancer-specific mortality (PCSM), compared to a neoadjuvant/concurrent approach [48]. Current clinical guidelines do not offer definitive recommendations regarding the optimal sequencing of ADT and EBRT. The AUA/ASTRO guidelines provide a conditional recommendation (evidence grade C), stating: “When combined ADT and radiation are used, ADT may be initiated neoadjuvantly, concurrently, or adjuvantly” [49]. Similarly, the ESTRO guidelines acknowledge that “it remains unclear whether intermediate-risk patients benefit more from neoadjuvant versus adjuvant ADT” [50]. Clinical practice remains variable, as illustrated by a recent national survey of Turkish radiation oncologists treating intermediate-risk prostate cancer: among those prescribing short-term ADT, 57% administered it concurrently with EBRT, while 43% favored a neoadjuvant approach [51]. However, the absence of clear clinical recommendation associated with the recent MARCAP findings may prompt a shift toward the concurrent/adjuvant strategy in clinical decision-making.

Recently, there has been a growing enthusiasm for focal EBRT dose escalation to the DIL, following the precedent set by the FLAME trial [13]. In FLAME, the DIL received a focal boost of up to 95 Gy in 35 fractions, while the whole prostate received 70 Gy. After a median follow-up of 72 months, the 5-year biochemical disease-free survival (bDFS) was significantly higher in the focal boost arm (92% vs 85%) without increased genitourinary or gastrointestinal toxicity or deterioration in quality of life [13]. Building on this, the multicenter phase II hypo-FLAME trial evaluated SBRT using 35 Gy in five weekly fractions with an *iso*-toxic focal boost up to 50 Gy, primarily in intermediate and high-risk patients. With a median follow-up of 61 months, the 5-year bDFS reached 93%, and rates of late grade ≥ 2 GU and GI toxicity were low (12% and 4%, respectively) [12]. Together, these trials demonstrate that MRI-guided focal dose escalation improves biochemical control without compromising safety, in both conventional and ultra-hypofractionated settings. However, none of these trials reported the timing of MRI relative to ADT initiation for patients receiving hormonal therapy. It is merely stated that “(neo)adjuvant hormonal therapy was prescribed in accordance with clinical practice”. Given that the mean DIL volume often ranges from 3 to 6 cc [52], initiating ADT and EBRT concurrently—especially in normofractionated regimens—can risk missing the correct boost volume due to ADT-induced volume reduction.

While the use of SBRT reduces the significance of prostate volume variability due to its shorter overall treatment duration, prostate volume can still decrease by more than 15% during the EBRT course if ADT is administered in a concurrent/adjuvant manner (Fig. 4). Moreover, the higher dose per fraction used in SBRT may amplify the clinical impact of a 15% prostate volume reduction on treatment-related toxicity. This concern was taken into account in the recent PACE-C trial, which recommended initiating EBRT within 12 weeks of starting ADT—preferably within 8 weeks—according to protocol guidelines [53]. Importantly, planning MRI was performed after ADT initiation but prior to CT simulation and EBRT delivery, allowing for accurate target delineation based on the downsized prostate. Finally, several studies have shown a significant increase in mean prostate volume during extreme hypofractionation without ADT [54]. Since SBRT alone increases prostate volume while ADT reduces it, MRI-Linac may represent the ideal technology for accurately delivering prostate SBRT. It allows for real-time adaptation to divergent effects of treatment components on prostate volume, enabling tighter PTV margins and reduced acute toxicity [55].

Due to the heterogeneity of available data, we focused specifically on prostate volume reduction and did not analyze the influence of pre-treatment variables or different hormonal regimens on the degree of shrinkage. However, several included studies in our systematic review examined the influence of different hormonal regimens on prostate volume reduction [22], [23], [26], [27], [28], [31], [34], [35], [42], [47]. Combined androgen blockade (LHRH agonist plus antiandrogen) resulted in slightly greater reductions than agonist monotherapy (±35% vs ± 30%)[22]. Similar trends were observed in a Japanese cohort, where mean reductions were 32% with LHRH agonists, 18% with antiandrogen monotherapy, and 41% with maximal androgen blockade [26]. When comparing LHRH agonists and bicalutamide monotherapy, no significant difference was observed (median reductions: 32% vs 26%)[27]. Additionally, 2 RCTs comparing LHRH antagonists (degarelix) and agonist-based regimens reported comparable volume reduction (36–39%) after 12 weeks [31], [34], although a third RCT suggested a marginally greater reduction with antagonists [35]. Two subsequent *meta*-analyses of these three RCTs confirmed that the choice between LHRH agonists and antagonists has minimal impact on prostate volume reduction [56], [57]. The modest differences in volume reduction between LHRH agonists, antagonists or combined androgen blockade (±5-10%) are unlikely to influence contouring or planning decisions. This reinforces that timing of initiation is a far more impactful parameter than choice of agent regarding dosimetric stability. Concerning pre-treatment variables, a retrospective study by Jetwah et al.[58], analyzed 189 prostate cancer patients who received neoadjuvant hormonal therapy prior to permanent prostate brachytherapy, aiming to determine the influence of these variables on prostate volume reduction. The median duration of neoadjuvant treatment was 4.9 months, and the mean prostate volume decreased from 62.5 cc to 39.0 cc, a 35.9% reduction. Multivariate regression identified initial prostate volume as the only significant predictor of both absolute and relative volume reduction, while other clinical variables (T stage, Gleason score, prostate-specific antigen − PSA, NCCN risk group) were not predictive after adjustment. In our dataset, we also observed a weak correlation between initial prostate volume and the extent of reduction (Fig. 3). However, heterogeneity in volume assessment methods across studies may have obscured stronger associations.

This systematic review, while offering valuable clinical insights into prostate volume kinetics under hormonal therapy, is limited by significant heterogeneity in imaging modalities, hormonal regimens, follow-up durations, and volume assessment methods across studies. The lack of standardized imaging protocols, combined with the predominance of retrospective designs and the absence of formal risk-of-bias assessment, constrains the robustness of conclusions. Key sources of bias include potential selection bias, inconsistent imaging and segmentation approaches contributing to measurement bias, and inadequate control of confounders such as baseline prostate volume and PSA. These factors may limit the accurate detection of volume change patterns. Moreover, the search was limited to the PubMed database, which may have resulted in omission of studies indexed exclusively in other databases such as Embase or Web of Science. Despite these limitations, the early rapid decline in prostate volume was consistently reported across studies regardless of imaging modality, hormonal regimen, or reporting method. This review therefore provides valuable clinical insights that should be considered when treating patients with EBRT. In the highly precise field of EBRT delivery, it is essential to account for fundamental aspects of prostate physiology, particularly the dynamic changes induced by hormonal treatment.

## 5 Conclusion

This systematic review shows that prostate volume reduction during hormonal treatment follows a predictable logarithmic decay, with the largest changes occurring within the first three months of treatment. These findings are supported by consistent trends across studies**,** although the overall certainty of evidence is moderate, primarily due to study heterogeneity, retrospective designs, and lack of standardized imaging protocols. These kinetics have implications for the sequence of hormonal treatment and EBRT. With evolving paradigms such as PSMA-guided staging, DIL-boosting and MRI-linear accelerator workflows, prostate cancer EBRT now depends on millimetric geometric precision. Understanding the non-linear kinetics of prostate volume reduction is essential to prevent geometric uncertainties that may become critical in SBRT of focal-boost protocols. Based on our analysis, the duration of hormonal therapy is a predictor of prostate volume reduction. Notably, early start of EBRT relative to hormonal treatment, particularly when combined with normofractionated regimens, may hinder optimal organ-at-risk sparing and target coverage due to dynamic volume changes. Current clinical practice varies widely, and major guidelines provide limited guidance on ADT sequencing. Future prospective studies are necessary to determine the best timing for EBRT planning and delivery in relation to ADT initiation, aiming to improve oncologic outcomes while reducing treatment-related toxicity.

## Data Availability Statement

This systematic review did not generate new primary data. All data analyzed during the review were extracted from published articles, which are cited in the manuscript. No additional datasets are available. The compiled extraction tables and synthesis files used in this study are available from the corresponding author upon reasonable request.

## Funding

none.

## Declaration of competing interest

The authors declare the following financial interests/personal relationships which may be considered as potential competing interests: Youssef Ghannam reports honoraria from MSD, recordati, ipsen, Bayer and travel funding from Accord Healthcare and MSD. David Pasquier reports consultancies and travel funding from Astellas, Accord, Bayer, Ipsen, Janssen and recordati. Mario Terlizzi reports consultancies and travel funding fromBayer, Ipsen, Astellas, Janssenm Accord Healthcare and Recordati. Vérane Achard reports honoraria and/or travel funding from Accord Healthcare, Astellas, Janssen, Medtis, Healthbook, Viatris. Bertrand Tombal reports grants or fundings from astellas, Bayer, Ferring, Myovant, Consultancies from Accord, Amgen, Astellas, AstraZeneca, Bayer, Ferring, Myovant, MSD, Novartis, Telix. Paul Sargos reports honoraria, consulting fees, and travel support from Astellas, Bayer, Janssen Sanofi, Accord-Healthcare, Ipsen, Ferring, Recordati, Novartis and Takeda.
